# Best Practices and Communication Strategies for Informing Oncology Patients About Treatment Discontinuation and Transition to Palliative Care—A Practical Guide for Oncologists

**DOI:** 10.3390/cancers17213566

**Published:** 2025-11-03

**Authors:** Aleksandra Piórek, Adam Płużański, Dariusz M. Kowalski, Maciej Krzakowski

**Affiliations:** Department of Lung Cancer and Thoracic Tumors, Maria Sklodowska-Curie National Research Institute of Oncology, 02-781 Warsaw, Poland; adam.pluzanski@nio.gov.pl (A.P.); dariusz.kowalski@nio.gov.pl (D.M.K.); maciej.krzakowski@nio.gov.pl (M.K.)

**Keywords:** oncology communication, breaking bad news, palliative care, treatment discontinuation, advance care planning, patient–physician relationship

## Abstract

**Simple Summary:**

Discontinuing active oncological treatment and initiating palliative care is one of the most complex challenges in cancer care. This narrative review provides oncologists with practical guidance for these difficult conversations. Drawing on clinical guidelines, scientific literature, and expert opinion, we propose a structured communication strategy and introduce a practical algorithm to support informed and compassionate decision-making. This article aims to help clinicians balance medical accuracy with empathy while respecting patient values and preferences.

**Abstract:**

Discontinuing active oncological treatment and initiating palliative care is a critical moment in cancer care, requiring oncologists to address complex clinical, ethical, and emotional challenges. This narrative review aims to provide clinicians with practical guidance for conducting conversations about treatment discontinuation and transitioning patients to palliative or hospice care. Drawing from current clinical guidelines, empirical research, and expert perspectives, the article reviews evidence-based communication strategies and frameworks, including the SPIKES protocol, Ask–Tell–Ask, the WHO model, and the disclosure model. The article also explores the clinical, functional, psychosocial, and ethical criteria relevant to treatment withdrawal decisions, as well as the timing and structure of end-of-life discussions. A practical algorithm is proposed, synthesizing key principles into a step-by-step guide for use in daily oncology practice. The algorithm supports clinicians in balancing medical indications with patient values and preferences, fostering shared decision-making and maintaining therapeutic relationships even in the most difficult circumstances. The review concludes that structured yet flexible communication enhances patient understanding, reduces unnecessary interventions, and improves the quality of end-of-life care. By promoting patient-centered care and timely palliative integration, this article offers oncologists a clear and adaptable approach to one of the most sensitive aspects of cancer care.

## 1. Introduction

In oncological practice, the decision to discontinue disease-directed therapy and transition to exclusively palliative care is among the most complex and emotionally charged moments for both patients and clinicians. It demands not only rigorous clinical judgment but also nuanced, empathetic communication that reflects patient preferences and integrates psychological, ethical, and systemic factors [1,2].

This narrative review synthesizes current best practices based on a comprehensive literature search conducted in the PubMed database. We used combinations of the following keywords: “oncology”, “cancer”, “communication”, “bad news”, “treatment discontinuation”, “palliative care”, “advance care planning”, and “end-of-life discussions.” The search covered English-language publications from January 2000 to June 2025. Additionally, we examined reference lists of relevant articles to identify further sources.

Priority was given to clinical guidelines, systematic reviews, and high-quality original studies focused on communication strategies in the context of transitioning from active oncologic treatment to palliative care. By integrating foundational and recent evidence, this review aims to provide oncologists with a practical, evidence-informed approach to navigating these critical conversations.

## 2. Criteria for Disqualification of an Oncology Patient from Disease-Directed Therapy

Discontinuing disease-directed therapy should be guided by a combination of clinical indicators, functional status, quality of life, and patient preferences. From a clinical standpoint, objective evidence of lack of efficacy—such as radiologic progression per RECIST 1.1 [3]—or serious treatment-related toxicity [4] may indicate that continuing therapy is not beneficial. Functional decline, as marked by poor performance status (ECOG ≥ 3) [5], presence of severe cachexia, or comorbidities, also supports re-evaluating treatment goals [1,3].

Furthermore, the impact of treatment on quality of life must be considered, especially if ongoing therapy contributes to physical suffering or psychological distress [4,6,7]. Finally, a patient’s explicit wishes and individual values are central. An informed decision to discontinue treatment, even in the absence of clear clinical futility, should be respected and supported [7,8,9]. Decisions must reflect a holistic perspective rather than isolated medical criteria [9].

## 3. The Timing of Initiating the Conversation

Determining the appropriate time to discuss treatment discontinuation requires balancing clinical judgment with sensitivity to the patient’s emotional readiness. Initiating the conversation too early may cause unnecessary anxiety; delaying it too long may deprive patients and families of the opportunity to prepare and make informed decisions [8,10,11]. Triggers such as radiologic or clinical progression, severe toxicity, or verbal and non-verbal cues from patients expressing uncertainty or shifting goals of care can help identify the right moment. Evidence suggests that early, well-structured discussions can enhance patient satisfaction and reduce distress [10].

## 4. Communication Models and Their Critique

Over the past two decades, several structured communication models have been developed to support clinicians in delivering difficult information, particularly during transitions from active treatment to palliative care. Among the most cited are the SPIKES protocol, the WHO model, the Ask–Tell–Ask technique, and the Individualized Disclosure Model. Although widely implemented, these models differ in structure, applicability, and outcomes.

### 4.1. Overview and Comparative Analysis

The SPIKES protocol [12] includes six sequential steps: Setting up the interview, assessing the patient’s Perception, obtaining an Invitation to share information, providing Knowledge, addressing Emotions, and formulating a Strategy. Its strength lies in its comprehensive nature and structured emotional support, making it suitable for discussions about diagnosis, recurrence, or treatment discontinuation. Studies have shown it improves clinician confidence and patient satisfaction.

The WHO model, while less detailed, offers an ethical framework emphasizing empathy, individualized delivery, and respect for the patient’s right to know. It serves well in cross-cultural settings where flexibility is critical [13]. However, its generality can lead to variability in application and outcome.

The Ask–Tell–Ask approach is a brief and adaptable strategy, encouraging bidirectional communication. Initially, the clinician asks what the patient understands, then provides tailored information, and concludes by verifying comprehension. This model is effective for routine updates or iterative discussions but may lack depth for emotionally charged conversations such as treatment cessation [10].

The Individualized Disclosure Model focuses on tailoring the timing, content, and style of communication to patient preferences and readiness [14]. It addresses the limitations of standardized approaches and aligns with evidence showing patient preferences vary based on age, culture, and emotional coping style.

While SPIKES and WHO protocols are better suited for initial and emotionally intense discussions, the Ask–Tell–Ask and Individualized Disclosure models excel in ongoing, adaptive dialogue. Despite their differences, no model comprehensively addresses the dynamic complexity of end-of-life oncology communication.

### 4.2. Flexible Adaptation in Practice

Real-world clinical scenarios often demand hybrid or adapted versions of these models. For instance, when dealing with patients with cognitive decline or severe anxiety, oncologists may condense SPIKES into three key elements: preparation, perception, and plan, while incorporating Ask–Tell–Ask for emotional calibration. An example comes from a Japanese study, where the SPIKES model was adjusted to accommodate collectivist cultural norms by involving family members early in the perception and invitation phases [15].

Similarly, in Southern Europe, where nondisclosure or partial disclosure remains common due to family pressure, the Individualized Disclosure Model offers a culturally sensitive alternative. Italian oncologists often negotiate communication content with both patient and family members, as highlighted by Grassi et al. (2000) [13].

### 4.3. Clinical Impact and Limitations

Although these models improve communication structure and provider comfort, empirical evidence on their impact on clinical outcomes is mixed. SPIKES-based training correlates with improved clinician self-efficacy and patient satisfaction but does not consistently reduce patient distress or decisional conflict [16]. Ask–Tell–Ask may enhance patient comprehension but shows limited influence on emotional processing. Individualized approaches appear more effective in aligning care with patient values but require significant clinician experience and flexibility.

In conclusion, communication in oncology benefits from structured models, yet none are universally sufficient. Effective practice requires situational awareness, cultural sensitivity, and adaptability. Future guidelines should integrate these dimensions and provide training for tailoring approaches to individual needs.

## 5. Breaking Bad News in Oncology—Practical Principles

Delivering bad news to oncology patients—whether about diagnosis, disease progression, or the transition from curative treatment to palliative care—is one of the most emotionally demanding tasks for clinicians. While communication models such as SPIKES, WHO, and Ask–Tell–Ask provide theoretical structure, their practical implementation requires adaptation to individual clinical contexts and patient preferences [12,13,17].

Preparation of the setting and timing is a foundational step, emphasized across all models. Conversations should occur in a private, quiet room with sufficient time to allow for emotional processing, free from interruptions or time pressure [4,12]. In practice, this means not only logistical planning, but also psychological readiness from the clinician to engage without distraction.

An essential early task is to assess the patient’s current understanding, in alignment with the “Perception” phase in SPIKES and the first “Ask” in Ask–Tell–Ask. Open-ended questions such as “What have you understood about your current condition?” help clinicians tailor the delivery of information to the patient’s cognitive and emotional state [12,17]. This step prevents misalignment between clinician intent and patient interpretation.

The gradual delivery of information, using the “chunk and check” strategy, is supported by all major models and supported by evidence indicating that cognitive overload can impair understanding [1,18]. Each piece of information should be delivered in small segments, interspersed with time for emotional reaction and clarification, enhancing retention and promoting trust.

Recognizing and responding to emotions is a core component of effective communication. The “E” step in SPIKES (Emotions) encourages empathetic reflection, such as acknowledging tears or silence with statements like “This seems very hard for you.” Clinical studies confirm that addressing emotions directly supports alliance and patient coping [1,4].

Maintaining hope without creating false expectations is a subtle but vital skill. Rather than offering unrealistic optimism, clinicians should reframe hope in terms of achievable goals—such as symptom relief, time with family, or maintaining autonomy [7,19]. This approach aligns with both the WHO model’s ethical emphasis and the evidence-based recommendation to balance truth and compassion [5,13].

When appropriate, involving family members enhances patient understanding and shared decision-making but must be done with the patient’s consent [20]. Clinicians should clarify who the patient wishes to be present and respect preferences about when and how to include relatives, a step especially crucial in cultures where family plays a primary role in medical decisions.

Documentation of the conversation ensures consistency across the care team and is essential for legal and clinical accountability. The note should include the content of the discussion, the patient’s emotional response, people present, and any decisions or follow-up plans [1].

Finally, these principles are most effective when integrated into a flexible communication approach that adjusts to the specific scenario. For example, SPIKES may be shortened in urgent care settings, while Ask–Tell–Ask is useful in outpatient clinics where discussions evolve over multiple visits. The WHO model’s focus on ethical communication is especially relevant in multicultural or transnational care settings [10,13]. Training programs for physicians in delivering bad news have a measurable impact on patient outcomes—in a randomized controlled trial, oncologists who underwent a two-day communication skills workshop based on patient preferences demonstrated significantly improved emotional support behaviors, and their patients showed a statistically significant reduction in depressive symptoms at follow-up [21].

## 6. Psychological and Ethical Aspects of Conversations

Conversations about treatment discontinuation and end-of-life care carry substantial psychological and ethical weight for both patients and clinicians. Effective communication must strike a balance between delivering truthful information and maintaining realistic hope. Research suggests that hope can be preserved when it is anchored in achievable goals, such as relief from distressing symptoms, improved comfort, or time with loved ones, rather than curative expectations in cases of incurable disease [22].

A particularly challenging dynamic is the phenomenon of collusion, where patients, families, and clinicians avoid open discussion of prognosis to protect each other from emotional distress. While well-intentioned, collusion can limit timely integration of palliative care, delay important decisions, and diminish the opportunity for patients to prepare emotionally and practically for the end of life [7].

Clinicians themselves face significant emotional burdens. Delivering bad news, particularly about the cessation of disease-directed treatment or impending death, can evoke feelings of failure, helplessness, and moral distress. Without adequate support, repeated exposure to such situations may lead to burnout or compassion fatigue [19]. To mitigate these effects, evidence-based strategies—such as participation in peer discussion groups, structured debriefings, or professional supervision—are recommended. Structured communication skills and emotional-support training programs for oncology clinicians have been shown to reduce burnout and increase satisfaction with patient interactions [23].

Ethical considerations are further complicated by cultural differences. In some societies, truth-telling is not automatically expected or desired by the patient; instead, families may prefer to shield patients from distressing information or take on the decision-making role. Oncologists must navigate these situations delicately, ensuring that the patient’s autonomy is respected while remaining culturally sensitive. This often requires early exploration of patient preferences and values regarding information disclosure and family involvement [13,14]. Ultimately, ethical communication requires self-awareness, cultural competence, and emotional resilience on the part of the clinician. Medical training programs should integrate these competencies alongside traditional communication protocols to better prepare physicians for complex, emotionally charged conversations at the end of life [19].

## 7. Advance Care Planning (ACP)

Advance Care Planning (ACP) is an ongoing, dynamic process that should be an integral component of comprehensive oncology care, particularly for patients with advanced disease or uncertain prognosis. The main objective of ACP is to help patients articulate their values, goals, and preferences regarding future medical care, especially in scenarios where their decision-making capacity may become compromised [6].

An “early” initiation of ACP refers to conducting these discussions before significant clinical deterioration—ideally at the point of diagnosis of advanced or incurable disease, or at the latest during the first signs of functional decline or increasing symptom burden [11]. Evidence suggests that early conversations are associated with greater alignment of care with patient values, reduced emotional distress, and decreased likelihood of receiving unwanted aggressive interventions at the end of life [6,11].

Key ACP discussions should explore patient priorities regarding life prolongation, quality of daily functioning, avoidance of burdensome or non-beneficial treatment, and the opportunity to spend meaningful time with loved ones. Clinicians should also ask about preferred setting of care at the end of life—whether home, hospice, or hospital—which supports appropriate planning and resource allocation.

A crucial element of ACP is formal documentation, which may include medical record notes, advance directives, or durable power of attorney for healthcare. These documents must be accessible to the entire medical team, and their content should be reviewed and updated periodically, particularly after major health status changes, hospitalizations, or patient preference shifts [6,11].

While ACP has clear clinical and ethical benefits, its implementation varies internationally due to legal, cultural, and systemic differences. In some countries, advance directives carry legal weight, while in others they serve only as recommendations. Therefore, oncologists should be aware of their local legal framework and, when necessary, involve legal or ethical consultants to ensure proper documentation and validity across care settings [6,9].

In practice, ACP enhances patient autonomy, reduces the burden on families, and supports clinicians in making ethically aligned decisions. Importantly, studies confirm that ACP does not reduce hope, but rather helps redirect it toward achievable goals—such as comfort, dignity, and control over the dying process [11].

Practical recommendations for ACP implementation:

- Initiate ACP discussions early, ideally upon diagnosis of advanced disease or functional decline;
- Explore patient values, care goals, and preferred location of end-of-life care;
- Ensure clear, accessible documentation and schedule regular reviews;
- Understand the local legal context and adapt documentation accordingly.

## 8. Barriers and Challenges for Physicians

Conversations about discontinuing disease-directed oncology treatment and transitioning to palliative care present substantial challenges for physicians, encompassing educational, emotional, and systemic dimensions. One of the most frequently cited barriers is the lack of formal communication training. Despite the recognized complexity of end-of-life conversations, many clinicians report acquiring these skills through observation rather than structured education. This gap can lead to avoidance of difficult topics or inadequate delivery of critical information, negatively impacting patient understanding and emotional readiness [4].

Another common barrier is physicians’ concern about diminishing hope. The fear of triggering despair or emotional collapse often results in delayed or overly softened messages. This protective instinct, while well-intentioned, may compromise the transparency necessary for patients and families to prepare effectively for care transitions. Studies indicate that clear, empathetic conversations—when grounded in patient values and delivered with emotional sensitivity—do not eliminate hope but instead reframe it toward realistic and meaningful goals [24,25].

Clinicians also face substantial emotional burdens themselves. Repeated exposure to death, suffering, and moral dilemmas can generate feelings of helplessness or failure, leading to burnout or compassion fatigue. Structured emotional support programs, peer discussions, and training workshops focused on managing uncertainty and emotional reactions have demonstrated positive effects on clinician well-being and communication performance. For example, a randomized controlled trial found that physicians who completed a two-day communication training workshop showed improved empathy and emotional support behaviors, while their patients reported lower depressive symptoms during follow-up visits [21].

Organizational constraints, particularly limited time and insufficient privacy, further hinder the ability to conduct meaningful conversations. Busy outpatient schedules, staff shortages, and lack of dedicated spaces for private discussions often force clinicians to deliver complex information in rushed or suboptimal conditions [1]. These system-level obstacles must be addressed through institutional changes that prioritize communication quality as an essential element of care delivery.

Addressing these challenges requires a multifaceted strategy. Integrating mandatory communication training into oncology curricula, establishing regular debriefings or supervision for emotional support, and allocating protected time for difficult discussions are critical steps. Institutions must recognize that high-quality communication is not an ancillary skill but a core competency that directly influences patient outcomes and clinician sustainability.

## 9. Scientific Evidence

Over the past two decades, numerous studies have examined communication practices with oncology patients in advanced stages of disease, including disclosure of prognosis, decision-making around discontinuing disease-directed therapy, and planning for future care.

One of the key sources is the systematic review by Hancock et al. (2007) [5], which analyzed 46 studies on truth-telling in prognosis discussions with patients diagnosed with advanced, incurable cancer. The authors found that, although most physicians express support for full disclosure, in practice they often avoid end-of-life discussions or provide selective information. The most frequently cited barriers include lack of formal communication training, fear of diminishing the patient’s hope, pressure from the patient’s family, time constraints in daily practice, and the physician’s own emotional discomfort.

Importantly, the review highlights that open, empathetic, and cognitively tailored conversations do not diminish hope; instead, they may improve patients’ understanding of their situation and enable better preparation for making decisions regarding subsequent care [5]. For instance, in the review by Hancock et al. (2007), more than half of the studies reported that patients who received open prognostic communication demonstrated improved alignment of care with their values and experienced less emotional distress [5].

A second important source is the position statement developed during the third European consensus meeting of experts in oncology communication, presented by Stiefel et al. (2018) [19]. This document emphasizes the need for a new generation of communication training programs that go beyond the mere knowledge of established protocols (such as SPIKES or ask–tell–ask) to include the development of physician self-awareness, the ability to manage one’s own emotions, skills in recognizing and responding to patient emotions, and the adaptation of communication strategies to the cultural context and individual needs of the patient.

The experts place particular emphasis on pivotal moments in cancer care—such as the discontinuation of disease-directed treatment and the transition to palliative care—which require the physician to be both substantively prepared to explain the rationale behind clinical decisions and emotionally resilient when faced with the reactions of patients and their families [19].

Both sources clearly indicate that high-quality communication in oncology requires the simultaneous integration of clinical, psychological, and ethical considerations, along with the continuous refinement of the physician’s interpersonal skills [5,19].

## 10. Limitations

This narrative review has several limitations. First, despite a comprehensive literature search, the review was limited to English-language publications, which may introduce geographic or cultural bias. Second, the reliance on narrative synthesis rather than systematic review methodology increases the risk of publication bias. Third, the applicability of the proposed algorithm may be limited in healthcare systems with differing legal frameworks, resource constraints, or cultural norms. Finally, while the communication models discussed are grounded in evidence, their real-world effectiveness can vary depending on clinician experience and patient population.

## 11. Conclusions

Conversations about treatment discontinuation and transitioning to palliative care remain among the most ethically complex and emotionally demanding aspects of oncology practice. This narrative review underscores the need for oncologists to go beyond clinical expertise and engage in flexible, structured, and emotionally attuned communication.

The comparative analysis of widely used models—SPIKES, WHO, Ask–Tell–Ask, and the Individualized Disclosure Model—reveals that no single framework is universally sufficient. Instead, clinicians must adapt strategies based on patient values, cultural context, emotional readiness, and clinical setting. Practical examples of such adaptation, as well as evidence on their varied clinical impacts, are essential for guiding real-world communication.

The proposed six-step algorithm (Table 1) integrates current clinical guidelines and empirical data with practical experience, offering a flexible yet structured approach for use in oncology settings. It addresses not only clinical indicators but also emotional dynamics, ethical dilemmas, and documentation requirements—supporting shared decision-making and therapeutic alliance.

High-quality communication improves patient understanding, reduces anxiety, prevents unwanted aggressive care, and aligns treatments with patient goals. However, this requires institutional support, ongoing training, and time allocation. Structured communication training has been shown to reduce physician burnout and enhance patient satisfaction and emotional outcomes [19,21].

Future work should focus on further validation of communication models in diverse populations and care settings, and on integrating cultural, systemic, and legal contexts into standardized protocols. Incorporating routine emotional support and supervision for clinicians, and addressing documented barriers such as time pressure or fear of diminishing hope, remain urgent priorities.

Effective end-of-life communication is not an ancillary task, but a core clinical skill that directly affects patient quality of life and ethical integrity in oncology care.

## Figures and Tables

**Table 1:** Practical Algorithm for Discontinuation of Active Oncology Treatment.

Step	Action	Description
1	Identify the clinical trigger	Use objective criteria indicating lack of treatment efficacy (e.g., disease progression, poor performance status).
2	Prepare for the conversation	Gather relevant clinical data, plan timing and setting to ensure a private, uninterrupted environment.
3	Establish the patient’s starting point	Determine the patient’s current understanding and their desire for further information.
4	Deliver information in stages	Communicate in small parts; observe and address emotional and cognitive reactions.
5	Discuss goals and care options	Present supportive, palliative, or hospice care aligned with patient values and clinical situation.
6	Document the agreements	Record the decisions, care plan, and schedule future conversations or follow-ups.
